# Predictive model of positive surgical margins after radical prostatectomy based on Bayesian network analysis

**DOI:** 10.3389/fonc.2024.1294396

**Published:** 2024-03-28

**Authors:** Guipeng Wang, Haotian Du, Fanshuo Meng, Yuefeng Jia, Xinning Wang, Xuecheng Yang

**Affiliations:** Department of Urology, The Affiliated Hospital of Qingdao University, Qingdao, China

**Keywords:** prostate cancer, positive surgical margin, magnetic resonance imaging, Bayesian network, radical prostatectomy (RP)

## Abstract

**Objective:**

This study aimed to analyze the independent risk factors for marginal positivity after radical prostatectomy and to evaluate the clinical value of the predictive model based on Bayesian network analysis.

**Methods:**

We retrospectively analyzed the clinical data from 238 patients who had undergone radical prostatectomy, between June 2018 and May 2022. The general clinical data, prostate specific antigen (PSA)–derived indicators, puncture factors, and magnetic resonance imaging (MRI) characteristics were included as predictive variables, and univariate and multivariate analyses were conducted. We established a nomogram model based on the independent predictors and adopted BayesiaLab software to generate tree-augmented naive (TAN) and naive Bayesian models based on 15 predictor variables.

**Results:**

Of the 238 patients included in the study, 103 exhibited positive surgical margins. Univariate analysis revealed that PSA density (PSAD) (*P* = 0.02), Gleason scores for biopsied tissue (*P* = 0.002) and the ratio of positive biopsy cores (*P* < 0.001), preoperative T staging (*P* < 0.001), and location of abnormal signals (*P* = 0.002) and the side of the abnormal signal (*P* = 0.009) were all statistically significant. The area under curve (AUC) of the established nomogram model based on independent predictors was 73.80%, the AUC of the naive Bayesian model based on 15 predictors was 82.71%, and the AUC of the TAN Bayesian model was 80.80%.

**Conclusion:**

The predictive model of positive resection margin after radical prostatectomy based on Bayesian network demonstrated high accuracy and usefulness.

## 1 Introduction

Radical prostatectomy is the most essential treatment for localized and locally advanced prostate cancer. However, due to the size and location of the tumor and the anatomical characteristics of the prostate, incomplete resection of the tumor may occur, resulting in positive surgical margins of pathological specimens (1). A positive surgical margin usually indicates a higher biochemical recurrence rate (2), and studies have shown that patients with positive surgical margins are 2–4 times more likely to experience biochemical recurrence than those with negative surgical margins (3) and that they also possess a potentially shortened progression-free survival (4). Therefore, if the probability of encountering a positive resection margin postoperatively can be effectively predicted prior to surgery, an appropriate treatment plan can be better formulated, and the surgical resection margin rate can then be reduced to slow the progression of the disease and improve patient prognosis.

Bayesian theory, which is a statistical theory corresponding to classical statistics, applies sample information to make inferences about a given population. The structure of a Bayesian network is a directed acyclic graph that represents the joint probability density between high-dimensional variables. The TAN Bayesian network (tree-augmented naive Bayesian network) is an extension of the classical Bayesian network model and can address correlated variables with favorable predictive ability for high-dimensional data. As a machine learning method combining probability theory and graph theory, Bayesian network can analyze the problem combined with conditional probability and is often used in disease prediction, treatment effect evaluation, diagnosis, and treatment decision making (5–7). On this basis, we established a predictive model for positive surgical margins after radical prostatectomy. We also calculated and analyzed the weight of each influencing factor to explore its clinical guiding significance.

## 2 Patients and methods

We collected data from patients who underwent transperitoneal laparoscopic or robot-assisted radical prostatectomy at the Affiliated Hospital of Qingdao University from June 2018 to May 2022. All patients underwent systematic 12-core biopsy and cognitive magnetic resonance imaging (MRI)/ultrasound fusion–targeted biopsy. We defined a positive postoperative resection margin as one where the tumor cells contacted the ink-stained edge of the surgical specimen; if the tumor cells were only close to the ink-stained edge, the surgical margin was regarded as negative. Each pathological section with a positive margin was evaluated by two independent pathologists, and the results were reviewed by the deputy director or higher of our department when there was any disagreement.

According to the postoperative pathological results, the patients were divided into a positive resection margin group and a negative resection margin group. The predictive variables of the two groups included general clinical factors (age, body mass index, prostate volume, and surgical type), PSA-derived indicators [total prostate-specific antigen (TPSA), the free (F)/TPSA ratio, PSA density (PSAD)]; biopsy factors (positive needle ratio, Gleason score); MRI-related factors [clinical stage, prostate imaging and reporting data system (PI-RADS) score, location of an abnormal signal, side of an abnormal signal, length of the membranous urethra (MUL), and maximum diameter of an abnormal signal]. The positive needle ratio was defined as the ratio of the number of pathologically positive needles to the total number of needles and was divided into three groups of <30%, 30%–60%, and ≥60%, and preoperative T staging was divided into T1–T2 and ≥T3 groups according to the 2017 AJCC tumor-staging criteria. Based upon the prostate imaging reports and data scoring system with 3.0T multi-parameter MRI, the PI-RADS scoring group was divided into four groups: 1, 3, 4, and 5. MUL was defined as the average distance from the tip of the prostate to the urethra at the level of the bulb of the penis in the mid-sagittal plane, and the maximal diameter of the abnormal signal was defined as the maximal diameter of the abnormal signal on the horizontal axis upon MRI T2WI.

## 3 Inclusion and exclusion criteria

### 3.1 Inclusion criteria

1. The postoperative pathological diagnosis was prostate cancer.
2. Radical prostatectomy was completed by laparoscopic or robot-assisted laparoscopic surgery, and the operators were all associate-chief physicians or above who had successfully completed their advanced training.

### 3.2 Exclusion criteria

1. The patient had undergone neoadjuvant endocrine therapy before radical surgery.
2. The original data were incomplete.
3. Postoperative pathology indicated benign prostatic tissue or prostatic intraepithelial neoplasia.

## 4 Statistical methods

We employed SPSS 26.0 statistical software to analyze our data. Measurement data with a normal distribution were presented as mean ± SD, and measurement data that did not follow a normal distribution were presented as median (interquartile range). The differences among groups were determined using the Kruskal–Wallis test. Counting data are expressed as frequencies, and we compared groups using the chi-squared test. The chi-squared test was used for univariate analysis of the above variables, and *P* < 0.05 was considered to be statistically significant. Logistic multivariable regression analysis was used to analyze the statistically significant factors, and *P* < 0.05 was considered statistically significant.

## 5 Development of predictive models

All data were initially divided into a test set and validation set according to an 8:2 ratio by random sampling, and these were then used to establish the predictive model and internal validation set, respectively. The TAN Bayesian model and naive Bayesian model based on 15 clinical predictors were established by exploiting the BayesiaLab software, and the R language was adopted to establish a nomogram model based on the independent prognostic factors for the calculation of accuracy. The respective receiver operating characteristic (ROC) curves of the Bayesian model and the nomogram model were constructed, and the advantages and disadvantages of the models were evaluated according to their areas under the ROC curves (AUCs). Finally, the BayesiaLab validation function was executed to perform *a priori* statistical analysis on the Bayesian model with high-predictive efficiency, and the positive margin was used as the target variable; the remaining variables were employed as the predictor variables for a posteriori analysis. Based on the results of the a posteriori analysis, we analyzed and calculated the Birnbaum importance measure and ranked the importance of each predictor variable (8).

## 6 Results

### 6.1 Characteristics of the included population

We reviewed the data from 238 patients in the present study. Among them, 192 underwent laparoscopic radical prostatectomy and 46 underwent robotic. We enrolled 103 patients with positive surgical margins postoperatively, with a positivity rate of 43.3%. There were 53 patients (51.5%) with apical positive margins, 12 patients (11.7%) with basal positive margins, and 38 patients (36.9%) with ≥2 positive margins. We noted 35 cases of biochemical recurrence in patients with positive surgical margins within one year, accounting for 34.0% (35 of 103). It has to be noted that 5.9% patients (eight of 135) manifested biochemical recurrence within 1 year after surgery. The median age was 67.94 ± 6.93 years, with a BMI of 25.19 ± 3.42; MUL of 12.10 ± 3.66; prostate volume of 76.8 (51.66–109.60); TPSA of 17.20 (10.08–35.19) ng/ml; F/TPSA of 0.11 (0.08–0.16); and PSAD of 0.24 (0.13–0.49) ng/ml. The median percentage of positive needles was 50% (17%–83%). The Gleason score was ≤6 in 41 cases (17.2%), 3 + 4 in 18 cases (7.6%), 4 + 3 in 32 cases (13.4%), eight in 44 cases (18.5%), and ≥9 in 103 cases (43.3%).

### 6.2 Correlation analysis of predictors

#### 6.2.1 Positive results of univariate analysis

Prognostic factors such as TPSA, PSAD, Gleason score of puncture pathology, ratio of positive needles, T stage, abnormal signal location, and abnormal signal side difference between positive and negative margin groups were statistically significant (*P* < 0.05) (**Table 1**).

**Table 1:** Univariate analysis between positive margin group and negative margin group.

Factors	Positive surgical margin (*n* = 103)	Negative surgical margin (*n* = 135)	*P*-value
Age			0.265
< 60	16 (15.5)	12 (8.9)	
60–75	72 (69.9)	99 (73.3)	
> 75	15 (14.6)	24 (17.8)	
BMI			0.740
< 18.5	5 (1.9)	4 (3.0)	
18.5–23.9	46 (44.5)	60 (44.4)	
> 23.9	52 (50.5)	71 (52.6)	
Prostate volume			0.411
≤ 50 ml	26 (25.2)	28 (20.7)	
> 50 ml	77 (74.8)	107 (79.3)	
Surgical methods			0.187
General laparoscopic surgery	81 (78.6)	96 (71.1)	
Robot-assisted laparoscopic surgery	22 (21.4)	39 (28.9)	
TPSA			0.008
< 4 ng/ml	9 (8.7)	9 (6.7)	
4–10 ng/ml	9 (8.7)	32 (23.7)	
10–20 ng/ml	29 (28.2)	43 (31.9)	
≥ 20 ng/ml	56 (54.4)	51 (37.7)	
F/TPSA			0.080
≤ 0.16	80 (79.2)	96 (71.6)	
> 0.16	21 (20.8)	38 (28.4)	
PSAD			0.020
≤ 0.15	21 (20.4)	46 (34.1)	
> 0.15	82 (79.6)	89 (65.9)	
Gleason score			0.002
≤ 6	9 (8.7)	32 (23.7)	
3 + 4	4 (3.9)	14 (10.4)	
4 + 3	16 (15.5)	16 (11.8)	
= 8	18 (17.5)	26 (19.3)	
≥ 9	56 (54.4)	47 (34.8)	
Ratio of positive needles			< 0.001
< 30%	40 (38.8)	89 (65.9)	
30%–60%	24 (23.3)	30 (22.2)	
≥ 60%	29 (37.9)	16 (11.9)	
T stage			< 0.001
T1–T2	84 (81.6)	128 (94.8)	
≥ T3	19 (18.4)	7 (5.2)	
Abnormal signal location			0.002
Peripheral zone	38 (36.9)	65 (48.1)	
Transition zone	40 (38.8)	59 (43.7)	
Multiple abnormal signals	25 (24.3)	11 (8.2)	
Abnormal signal side			0.009
Left side	29 (28.2)	63 (46.7)	
Right side	38 (36.9)	44 (35.6)	
Bilateral	36 (34.9)	28 (20.7)	
PI-RADS score			0.098
1	1 (1.0)	0 (0)	
3	10 (9.7)	24 (17.8)	
4	44 (42.7)	64 (47.4)	
5	48 (46.6)	47 (34.8)	
Maximum transverse diameter of abnormal signal			0.053
< 12 mm	36 (35.0)	53 (39.3)	
12–21 mm	24 (23.3)	45 (33.3)	
> 21 mm	43 (41.7)	37 (27.4)	
MUL			0.358
≤ 10 mm	35 (34.0)	44 (32.6)	
10–13 mm	41 (40.0)	51 (37.8)	
> 13 mm	27 (26.2)	40 (29.6)	

#### 6.2.2 Results of logistic multivariable regression analysis of positive surgical margin

We conducted multivariable analysis on the indicators with statistically significant differences in the univariate analysis, and our results revealed that T stage and positive needle ratio were independent predictors of positive resection margin after prostate cancer surgery (**Table 2**).

**Table 2:** Multivariable analysis of postoperative margin-positive and -negative groups.

Factors	B	Standard deviation	OR	95% CI	*P*-value
	T stage				0.006
T1–T2	1		1		
≥ T3	1.274	0.488	3.574	1.975–9.298	
	Positive needle ratio				< 0.001
< 30%	1		1		
30%–60%	0.609	0.339	1.839	0.947–3.573	
≥ 60%	1.618	0.359	5.043	2.496–10.189	

### 6.3 Development of predictive models

#### 6.3.1 Naive Bayesian network model and effectiveness evaluation

**Figure 1:**
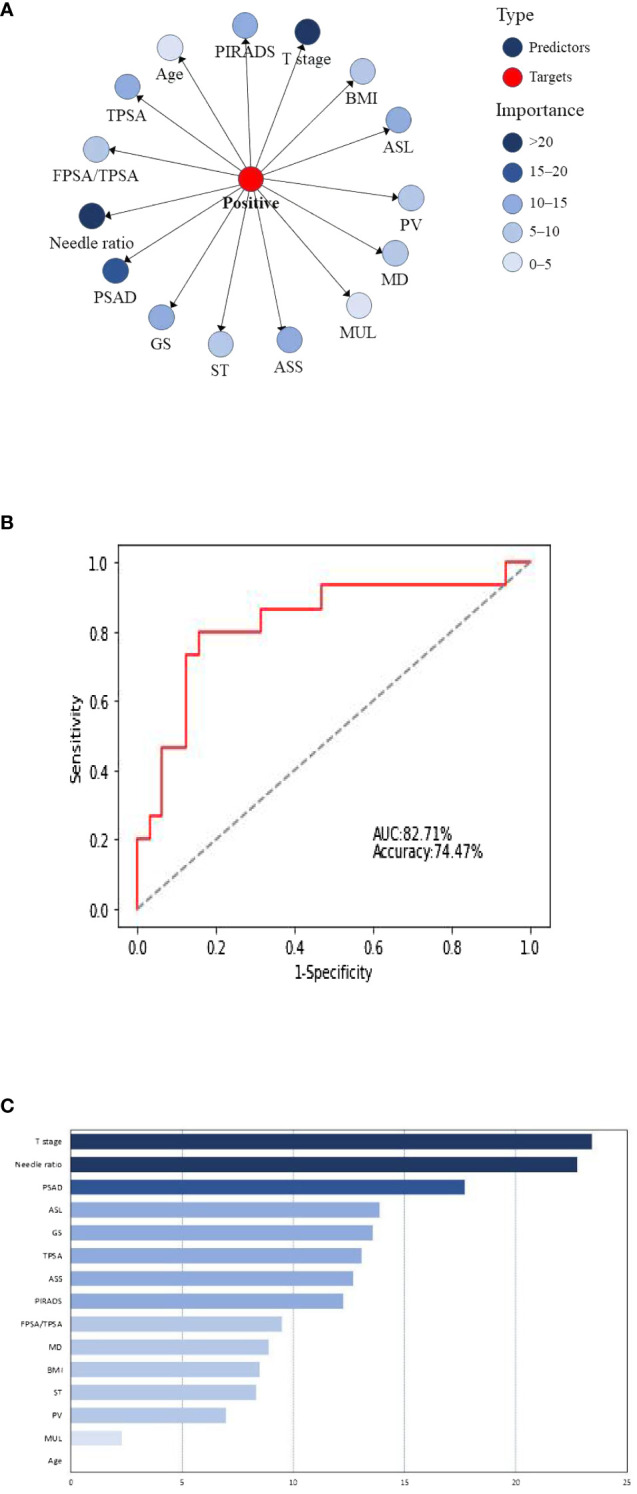
**(A)** Naive Bayesian model for predicting positive surgical margins after radical prostatectomy. BMI, body mass index; ASL, abnormal signal location; PV, prostatic volume; MD, maximum diameter of abnormal signal; Mul, length of the membranous urethra; ASS, abnormal signal side; ST, surgery type; GS, Gleason score. **(B)** Naive Bayes ROC curve. **(C)** Naive Bayesian significance analysis.

The 15 clinical predictors in **Table 1** were included to establish a naive Bayesian prediction model (**Figure 1A**). In this figure, the red nodes represent the target value, the blue nodes indicate the predicted value, the depth of the color designates its importance, and the darker the color the higher the importance. The ROC curve was established using the model data (**Figure 1B**), and the AUC of the model was 81.43%. We applied the BayesiaLab verification function to analyze, calculate, and rank the importance of the model, and the results of importance ranking showed that clinical stage and positive needle ratio were in the first importance interval, and that PSAD was in the second importance interval, with an interval of 15–20. Location of an abnormal signal, Gleason score, TPSA, side of abnormal signal, and PI-RADS score were in the third interval, with important intervals of 10–15 (**Figure 1C**).

#### 6.3.2 TAN Bayesian network model and effectiveness evaluation

**Figure 2:**
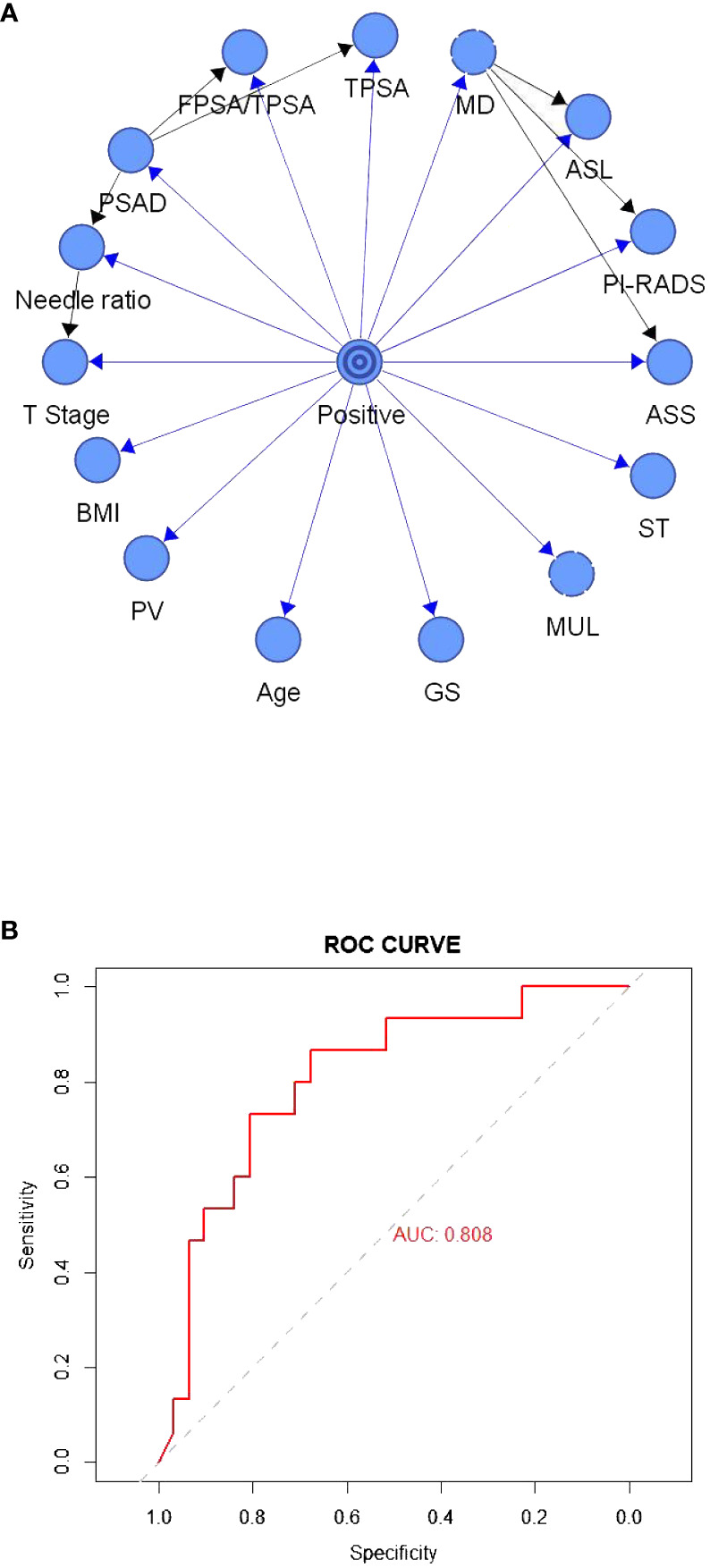
**(A)** The tree-augmented Bayesian prediction model. **(B)** ROC curve of the tree-augmented Bayesian prediction model.

**Figure 2A** depicts the TAN Bayesian prediction model based on the 15 clinical predictors in **Table 1**, with the central node representing the target value and the peripheral node representing the predicted value. The ROC curve was established based on the model data (**Figure 2B**), and the AUC of this model was 80.80%. In the TAN Bayes model variables, PSAD was closely related to F/TPSA, TPSA, and positive needle ratio; the positive needle ratio was correlated with T stage, and the maximum transverse diameter of the tumor was correlated with abnormal signal location, abnormal signal side and PI-RADS score.

#### 6.3.3 Nomogram model and efficiency evaluation

**Figure 3:**
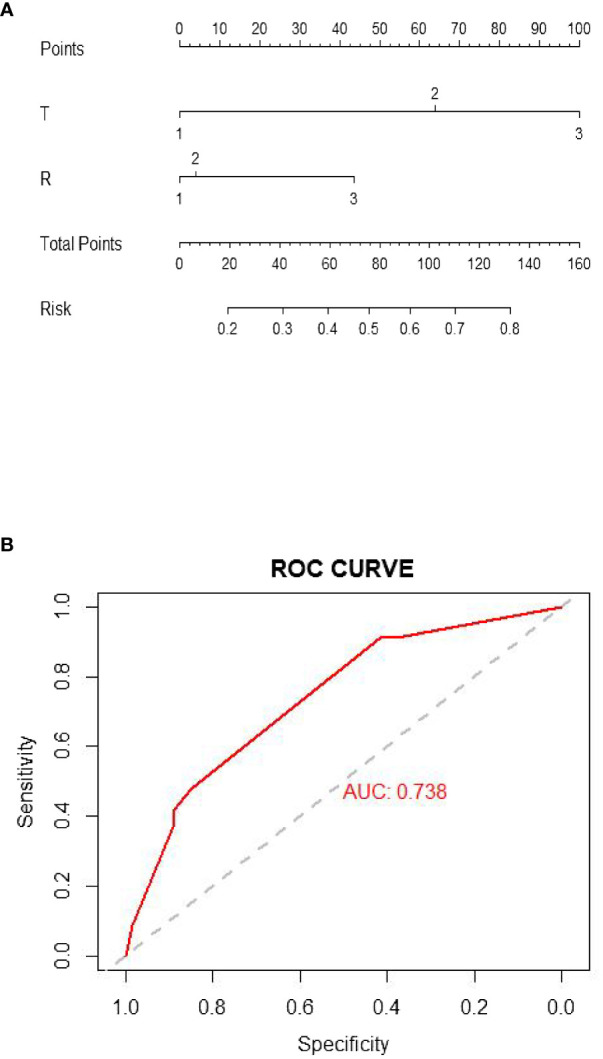
**(A)** The nomogram model. **(B)** ROC curve of the nomogram model.

The nomogram model that we constructed was based on independent prognostic factors in the multivariate analysis as shown in **Figure 3A**, and the established ROC is shown in **Figure 3B**, with an AUC of 73.80%.

## 7 Discussion

Radical prostatectomy is one of the preferred treatment options for early localized and partial locally progressive prostate cancer (9). Its goal is to eradicate prostate tumors, control disease progression, and ensure urinary control and sexual ability as much as possible, and to improve patients’ quality of life. Most prostate cancer patients can achieve a clinical cure after radical prostatectomy, but postoperative complications and tumor recurrence can significantly affect quality of life and even the lifespan of patients (10, 11). A positive surgical margin is an important predictor of poor prognosis, and studies have revealed that patients with a positive surgical margin show a significantly increased risk of biochemical recurrence and even of tumor progression (12). In the present study, 35 patients with positive surgical margins manifested biochemical recurrence within one year, accounting for 34.0% (35 of 103), and eight patients (5.9%, eight of 135) with negative surgical margins had biochemical recurrence within 1 year. The probability of biochemical recurrence in our patients with positive surgical margins was 5.7 times higher than in patients with negative surgical margins, confirming our analysis. In addition, patients with positive surgical margins require further adjuvant therapy (13) (such as local radiotherapy and endocrine therapy), and carry increased psychological burdens (14). Local treatment may prolong recovery time with respect to urinary control and complications such as radiation proctitis that affect patient quality of life (15). Therefore, if positive surgical margins can be effectively identified before surgery, corresponding treatment strategies can be better formulated, and the surgical margin rate can then be reduced, which will slow the progression of the disease and improve the overall quality of life of patients after surgery.

In view of the adverse effects of a positive surgical margin on prognosis, the creation of a model that clinicians can use to evaluate the risk of positive surgical margins and the benefits of surgery is particularly important. A model that predicts early radical prostatectomy for patients with a low probability of positive surgical margins will engender improved surgical benefit. For patients with a high probability of a positive surgical margin, preoperative neoadjuvant endocrine therapy can reduce the volume of the prostate tumor, reduce the tumor stage, and allow for appropriate timing of surgery according to patient condition; this will, in turn, reduce the probability of a positive surgical margin and enhance the achievement of a favorable radical treatment effect. Nomogram, as a commonly used clinical prediction model, can transform complex regression equations into simple visual graphs. Chinese researchers have previously generated a nomogram for the risk of positive surgical margins in prostate cancer based on preoperative factors, and it has been confirmed to exhibit acceptable predictive value in later stages (16). However, nomograms also have their limitations. When a nomogram model contains too many predictors, it is prone to overfitting and, thus, subject to strict conditions. For example, the dependent variables included in the logistic regression model should follow an exponential distribution, while the establishment of regression models is primarily based on statistically significant factors. Bayesian network analysis, as a machine learning method combining probability theory with graph theory, can be used to analyze a problem structure combined with conditional probability, and it is often implemented in the establishment of models such as disease prediction (17), treatment effect evaluation (7), and diagnosis and treatment decision making (18)—importantly, it displays acceptable efficiency. In this study, analyses by naive Bayesian network, TAN Bayesian network, and a nomogram model were exploited to predict positive surgical margins after radical prostatectomy, with the respective ROC curves for the Bayesian network and nomogram model constructed and the AUCs calculated to evaluate the superiority or inferiority of the models. Our results showed that the AUC for the naive Bayes model based on 15 predictors was 81.43%, which was higher than the 80.8% for the TAN Bayesian network model and the 69.2% for the nomogram model based on the same dataset, thus reflecting good predictive efficiency. We hypothesize that the high-predictive level was due to the following two reasons: the construction of the Bayesian network was not only limited to independent predictors but also included non-independent predictors that exerted a greater impact on the outcome. Although some predictors are not independent predictors, they still generate a certain impact on the results. Therefore, only by integrating as many factors as possible that exert an impact on the results can we achieve a prediction that is closest to the actual situation. However, Bayesian network analysis also allows for a small number of missing data and adopts relevant algorithms to infer data so as to avoid sample size and accuracy reductions caused by a small number of missing data.

In addition, TAN Bayesian network models allow associations to be uncovered between predictor variables and do not only show relationships between the predictor variable and the target variable but also reveal the corresponding relationships between the prediction variables (19). This compensates for the shortcomings of the previous classical statistical models, where it was difficult to resolve complex relationships between multiple variables. In this study, PSAD was found to be closely related to F/TPSA, TPSA, and the positive needle ratio; the maximum transverse diameter of the tumor was correlated with abnormal signal location, abnormal signal side, and PI-RADS score. In contrast to the TAN Bayesian network, the naive Bayesian model only facilitates the study of the relationship between the predictor variables and the target variables, and the default predictor variables reflect no correlation. The correlation between predictor variables herein was small, such that the predictive performance of the naive Bayesian model was higher than that of the TAN Bayesian model. In practice, different Bayesian models can be selected according to correlations between predictor variables so as to achieve optimal predictive ability (20).

With the continuous progress demonstrated in science and technology, an increasing number of machine learning algorithms have been applied to research on clinical issues (15). Computer algorithms can be applied to mine the relationships between clinical data by themselves, and this can compensate for the shortcomings of summarization through past self-experiences and, thus, provide a simpler and more efficient way to solve clinical problems (21–23). This study was an attempt to resolve a problematic clinical issue with a computer algorithm and our model proved successful and highly accurate. Through this model, we could then accurately predict the risk of postoperative positive surgical margins in patients. We recommend that early radical prostatectomy be performed in patients at low risk so as to improve the surgical benefit in patients. There were also some limitations to the current study due to its relatively small sample size and its design as a single-center study, and the predictive impact of the model on disparate populations was still unclear without external validation. However, we posit that, with the addition of a large amount of multi-center data and the continuous improvement of models using machine learning, the Bayesian prediction model of positive surgical margins after radical prostatectomy can provide a robust basis for clinical decision making.

## Data availability statement

The raw data supporting the conclusions of this article will be made available by the authors, without undue reservation.

## Ethics statement

The studies involving humans were approved by the Ethics Committee of the Affiliated Hospital of Qingdao University. The studies were conducted in accordance with the local legislation and institutional requirements. Written informed consent for participation was not required from the participants or the participants’ legal guardians/next of kin in accordance with the national legislation and institutional requirements.

## Author contributions

GW: Writing – original draft. HD: Data curation, Software, Writing – original draft. FM: Data curation, Formal analysis, Writing – original draft. YJ: Conceptualization, Methodology, Supervision, Writing – review & editing. XW: Methodology, Writing – review & editing. XY: Conceptualization, Writing – review & editing.

## References

[1] Tewari A Sooriakumaran P Bloch D Seshadri-Kreaden U Hebert A Wiklund P. Positive surgical margin and perioperative complication rates of primary surgical treatments for prostate cancer: a systematic review and meta-analysis comparing retropubic, laparoscopic, and robotic prostatectomy. Eur Urol. (2012) 62(1):1–15. doi: 10.1016/j.eururo.2012.02.029 22405509

[2] Ozbek A Ozbek R Duvarci M Kandemir O. Does the distance of the tumor from the surgical margin affect biochemical recurrence in patients with pathological organ-confined prostate cancer? Turk Patoloji Dergisi. (2021) 37:233–8. doi: 10.5146/tjpath.2021.01546 PMC10510620 34514575

[3] Matti B Reeves F Prouse M Zargar-Shoshtari K. The impact of the extent and location of positive surgical margins on the risk of biochemical recurrence following radical prostatectomy in men with Gleason 7 prostate cancers. Prostate. (2021) 81:1428–34. doi: 10.1002/pros.24240 34570379

[4] Swindle P Eastham JA Ohori M Kattan MW Wheeler T Maru N. Do margins matter? The prognostic significance of positive surgical margins in radical prostatectomy specimens. J Urol. (2008) 179:S47–51. doi: 10.1016/j.juro.2008.03.137 18405751

[5] Geng ZM Cai ZQ Zhang Z Tang ZH Xue F Chen C. Estimating survival benefit of adjuvant therapy based on a Bayesian network prediction model in curatively resected advanced gallbladder adenocarcinoma. World J Gastroenterol. (2019) 25:5655–66. doi: 10.3748/wjg.v25.i37.5655 PMC6785523 31602165

[6] Fenton N Neil M. Comparing risks of alternative medical diagnosis using Bayesian arguments. J Biomed Inform. (2010) 43(4):485–95. doi: 10.1016/j.jbi.2010.02.004 20152931

[7] Trilla-Fuertes L Gámez-Pozo A Arevalillo JM López-Vacas R López-Camacho E Prado-Vázquez G. Bayesian networks established functional differences between breast cancer subtypes. PloS One. (2020) 15:e0234752. doi: 10.1371/journal.pone.0234752 32525929 PMC7289386

[8] Friedman N Geiger D Goldszmidt M. Bayesian network classifiers. Mach Learn. (1997) 29:131–63. doi: 10.1023/A:1007465528199

[9] Costello AJ. Considering the role of radical prostatectomy in 21st century prostate cancer care. Nat Rev Urol. (2020) 17:177–88. doi: 10.1038/s41585-020-0287-y 32086498

[10] Zhang-Yin J Montravers F Montagne S Hennequin C Renard-Penna R. Diagnosis of early biochemical recurrence after radical prostatectomy or radiation therapy in patients with prostate cancer: State of the art. Diagn Interventional Imaging. (2022) 103:191–9. doi: 10.1016/j.diii.2022.02.005 35227633

[11] Clements MB Tin AL Estes CL Jibara G Desai PK Ehdaie B. Characterization of symptoms after radical prostatectomy and their relation to postoperative complications. J Urol. (2022) 207:367–74. doi: 10.1097/JU.0000000000002202 PMC9172597 34544264

[12] Oberlin DT Casalino DD Miller FH Matulewicz RS Perry KT Nadler RB. Diagnostic value of guided biopsies: fusion and cognitive-registration magnetic resonance imaging versus conventional ultrasound biopsy of the prostate. Urology. (2016) 92:75–9. doi: 10.1016/j.urology.2016.02.041 PMC4882086 26966043

[13] Thompson IM Tangen CM Paradelo J Lucia MS Miller G Troyer D. Adjuvant radiotherapy for pathological T3N0M0 prostate cancer significantly reduces risk of metastases and improves survival: long-term followup of a randomized clinical trial. J Urol. (2009) 181:956–62. doi: 10.1016/j.juro.2008.11.032 PMC3510761 19167731

[14] Punnen S Cooperberg MR D'Amico AV Karakiewicz PI Moul JW Scher HI. Management of biochemical recurrence after primary treatment of prostate cancer: a systematic review of the literature. Eur Urol. (2013) 64:905–15. doi: 10.1016/j.eururo.2013.05.025 23721958

[15] Abugharib A Jackson WC Tumati V Dess RT Lee JY Zhao SG. Very early salvage radiotherapy improves distant metastasis-free survival. J Urol. (2017) 197:662–8. doi: 10.1016/j.juro.2016.08.106 27614333

[16] Cheng W. Establishment and validation of nomogram for positive surgical margin of prostate cancer. Chin J Urol. (2020) 03):205–9. doi: 10.3760/cma.j.cn112330-20190821-00378

[17] Moul JW Connelly RR Lubeck DP Bauer JJ Sun L Flanders SC. Predicting risk of prostate specific antigen recurrence after radical prostatectomy with the Center for Prostate Disease Research and Cancer of the Prostate Strategic Urologic Research Endeavor databases. J Urol. (2001) 166:1322–7. doi: 10.1016/S0022-5347(05)65761-8 11547066

[18] Huang R Jiang L Cao Y Liu H Ping M Li W. Comparative efficacy of therapeutics for chronic cancer pain: A bayesian network meta-analysis. J Clin Oncol Off J Am Soc Clin Oncol. (2019) 37:1742–52. doi: 10.1200/JCO.18.01567 PMC6638598 30939089

[19] Jarrett D Stride E Vallis K Gooding M. Applications and limitations of machine learning in radiation oncology. Br J Radiol. (2019) 92:20190001. doi: 10.1259/bjr.20190001 31112393 PMC6724618

[20] Stish BJ Pisansky TM Harmsen WS Davis BJ Tzou KS Choo R. Improved metastasis-free and survival outcomes with early salvage radiotherapy in men with detectable prostate-specific antigen after prostatectomy for prostate cancer. J Clin Oncol Off J Am Soc Clin Oncol. (2016) 34:3864–71. doi: 10.1200/JCO.2016.68.3425 27480153

[21] Orvieto MA Alsikafi NF Shalhav AL Laven BA Steinberg GD Zagaja GP. Impact of surgical margin status on long-term cancer control after radical prostatectomy. BJU Int. (2006) 98:1199–203. doi: 10.1111/j.1464-410X.2006.06563.x 17125478

[22] Peiffer-Smadja N Rawson TM Ahmad R Buchard A Georgiou P Lescure FX. Machine learning for clinical decision support in infectious diseases: a narrative review of current applications. Clin Microbiol Infection Off Publ Eur Soc Clin Microbiol Infect Dis. (2020) 26:584–95. doi: 10.1016/j.cmi.2019.09.009 31539636

[23] Eraslan G Avsec Ž Gagneur J Theis FJ. Deep learning: new computational modelling techniques for genomics. Nat Rev Genet. (2019) 20:389–403. doi: 10.1038/s41576-019-0122-6 30971806

